# Anti-Proliferative Activity of HPOB against Multiple Myeloma Cells via p21 Transcriptional Activation

**DOI:** 10.3390/molecules23051044

**Published:** 2018-04-30

**Authors:** Linlin Liu, Xiaoyang Sun, Yu Xie, Yinping Zhuang, Ruosi Yao, Kai Xu

**Affiliations:** 1 College of Medical Imaging, Xuzhou Medical University, Xuzhou 221004, Jiangsu, China; liulinlin@xzhmu.edu.cn (L.L.); zyp-xz@163.com (Y.Z.); 2 Blood Diseases Institute, Xuzhou Medical University, Xuzhou 221004, Jiangsu, China; 100002015027@xzhmu.edu.cn (X.S.); 15952155422@163.com (Y.X.)

**Keywords:** HPOB, proliferation, apoptosis, p21, multiple myeloma

## Abstract

Histone acetylation or deacetylation is closely associated with the progression of multiple myeloma (MM). Currently, many histone deacetylase (HDAC) inhibitors have been approved for being used in clinical trials, but theirtherapeutic effectsarestill not ideal. As a novel HDAC inhibitor, hydroxamicacid-based small-molecule *N*-hydroxy-4-(2-[(2-hydroxyethyl)(phenyl)amino]-2-oxoethyl)benzamide (HPOB)’s possible roles in MM have not been studied. In this present study, the effect of HPOB as a potential anti-tumor agent in preventingproliferation and inducing apoptosis of MM cells had been investigated in detail. Our results showed that HPOB decreased the survival of MM cells in dose- and time-dependent manner. In addition, HPOB caused the accumulation of MM cells in G1 phase compared with the dimethylsulfoxide (DMSO) control group. Interestingly, we found that HPOB could overcome bortezomib (BTZ) resistance inMM cells and combining HPOB with BTZ could further sensitize MM cells. Certainly, our data illuminated that HPOB-mediated cell death occurs via transcriptional activation of p21, which was associated with an elevated level of global histone 3 acetylation (H3Ac) modification. Therefore, HPOB could be a potential candidate for MM treatment and the combination of HPOB and bortezomibcould bea possible therapeutic strategy for relapsed and refractory MM.

## 1. Introduction

Multiple myeloma (MM) is one of the most frequent hematological tumors in the world and is characterized by clonal proliferation of the plasma cells associated with elevated serum monoclonal proteins [1,2]. In the past few years, many novel therapeutic agents have been introduced in the treatment for MM patients, such as bortezomib, thalidomide or lenalidomide [3,4,5]. However, multiple myeloma remains incurable dueto its resistance against chemotherapy. Therefore, it is urgent to illuminate the molecular mechanism of MM progression and pursue more effective targeted molecular drugs.

Histone deacetylase (HDAC) inhibitors have a crucial role in the epigenetic regulation of gene expression mediating cancer cell survival and progression. To date, a large number of HDAC inhibitors have been generated from natural or artificial sources, which belong to a novel class of anti-tumor drugs targeting the HDACs involved in many signaling pathways [6,7]. Among them, panobinostathasbeen studied in various tumors as a single agent and also exhibited considerableanti-cancer activities when combined with other therapeutic agents [8,9,10]. Oralpanobinostat had been approved for use in combination with bortezomib and dexamethasone in patients with relapsed or refractory MM in many countries. However, the cytotoxicity of panobinostatwas found to increasesignificantly when used in combination with bortezomib, dexamethasone or lenalidomide [11,12]. Therefore, the development of novel selective HDAC inhibitors will contribute to the exploration ofthe huge therapeutic potential of HDAC inhibitors.

HPOB is a novel selective HDAC6 inhibitor, which inhibits the growth of multiple cancer cells and enhances cell death, although it does not have these effects on normal cells [13]. Subsequently, it has been reported that HPOB might be a potential target forblocking the glucocorticoid-mediated cell apoptosis [14]. Recently, Li et al. reported that HPOB also exhibited selective inhibition in Acute Myelocytic Leukemia (AML) cells [15]. It is currently unclear whether HPOB is involved in MM progression. In this study, we found that the HDAC inhibitor HPOB dose-dependently inhibited MM cell viabilities. Furthermore, HPOB could lead to a G1 phase arrest in the MM cell cycle. Additionally, HPOB induced MM cell apoptosis via the transcriptional activation of p21 together with an elevated H3Ac modification level. In conclusion, our data pavethe way for the development of novel molecular targeted therapeutic drugs formultiple myeloma.

## 2. Results and Discussion

### 2.1. HPOB Suppressed the Proliferation of MM Cells

**Figure 1:**
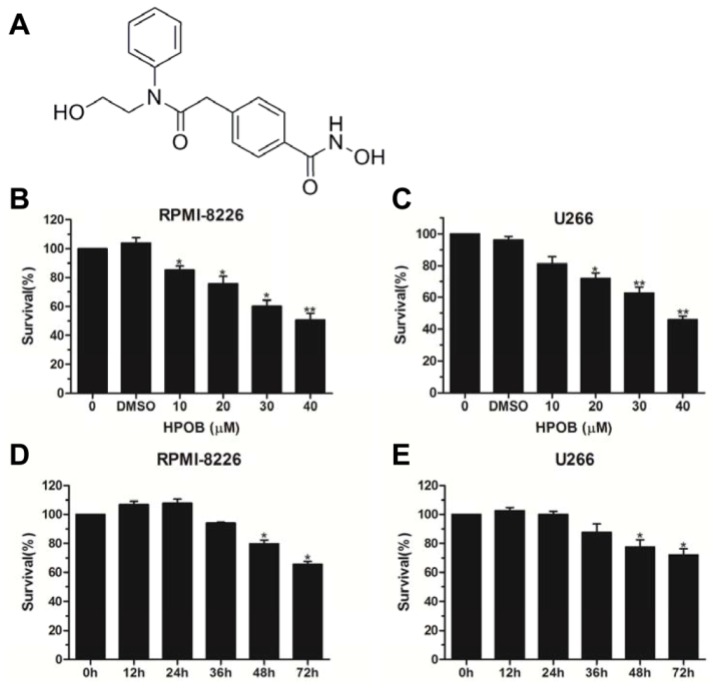
HPOB suppressed the proliferation of MM cells. (**A**) The molecular structure of HPOB; As indicated, theRPMI-8226 (**B**) and U266 (**C**) cells treated by different concentrations of HPOB for 48 h were analyzed by the CCK8 assay; (**D**,**E**) RPMI-8226 and U266 cells were treated by 40 μM of HPOB for different periods of time and analyzed by the CCK8 kit.

To explore the influence of HPOB on the cytotoxicity of MM cells (Figure 1A), we used a Cell Counting Kit-8 (CCK8) cell viability assay. RPMI-8226 and U266 cell lines derived from peripheral blood were used to conduct the in vitro experiments, which mimicked myeloma diseases. As shown in Figure 1B,C, HPOB treatment led to an obvious decrease in the RPMI-8226 and U266 cell viabilities in a dose-dependent manner. Furthermore, we wanted to know whether HPOB time-dependently inhibited cell proliferation. Unfortunately, the inhibition of cell viability was only observed following a 48-h and 72-h exposure to 40 μM of HPOB in RPMI-8226 and U266 cells (Figure 1D,E). Based on these data, we considered that the HDAC inhibitor HPOB treatment decreased MM cell survival.

### 2.2. HPOB Induced Cell Cycle G1 Phase Arrest

**Figure 2:**
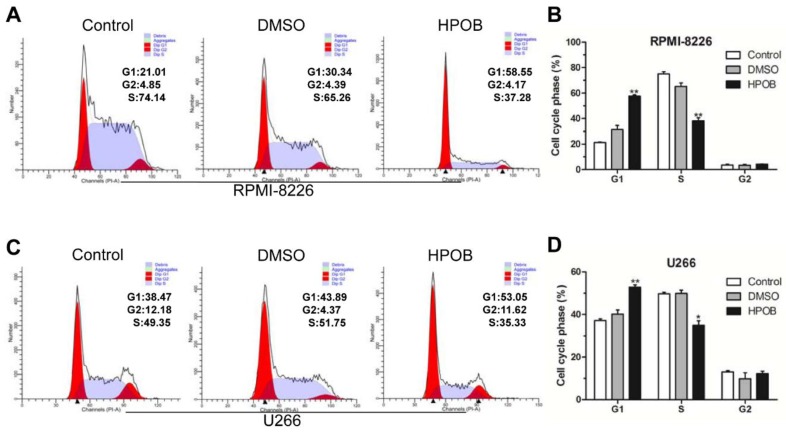
HPOB induced G1 phase arrest in thecell cycle. Cell cycle distribution was obtainedby PI staining and flow cytometry analysis in RPMI-8226 (**A**,**B**) and U266 (**C**,**D**) cells following treatment with 40 μM of HPOB for 48 h. Error bars indicate mean ± SD. * *p* < 0.05, ** *p* < 0.01.

After this, we investigated whether HPOB disrupted the cell cycle. Based on the results of the CCK8 assay, HPOB had a considerable inhibitory effect on the MM cell lines in a dose- and time-dependent manner. Flow cytometry analysis indicated that the HPOB in a lower concentration did not lead to cell cycle arrest (data not shown). When treated with 40 μM of HPOB for 48 h, a higher percentage of RPMI-8226 cells were found to bein the G1 phase and a lower percentage in the S phase, compared with the DMSO control group (Figure 2A,B). Additionally, HPOB treatment also increased the percentage of cells in the G1 phase in U266 cells compared with the DMSO control group (Figure 2C,D). In summary, the cell cycle was significantly blocked at the G1 phase in MM cells.

### 2.3. Effects of HPOB Treatment on Cell Apoptosis

**Figure 3:**
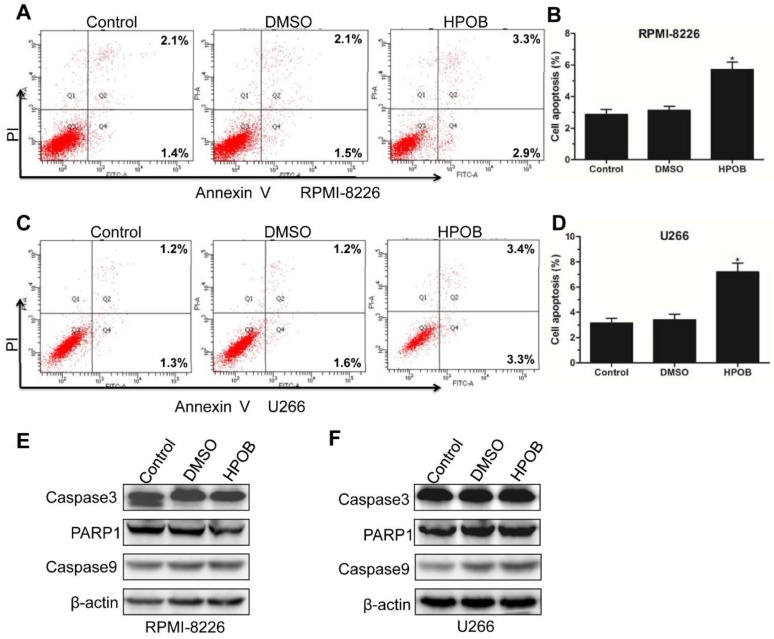
HPOB induced the MM cell apoptosis. Annexin V-FITC/PI staining and flow cytometry analysis shows the ratio of apoptotic RPMI-8226 (**A**,**B**) and U266 (**C**,**D**) cells treated by 40 μM of HPOB for 48 h; (**E**,**F**) Western Blot analysis of apoptosis-related proteins, withβ-actin used as an internal control. Error bars indicate mean ± SD. * *p* < 0.05.

Cell apoptosis is closely related with the cell cycle, which is a normal physiological process formaintaining cell homeostasis [16]. Many compounds inducing cell cycle arrest and apoptosis also canbe potential anti-tumor agents [17,18,19]. A lower concentration of HPOB was unable to induce MM cell apoptosis-related morphological changes (data not shown). Annexin V-FITC/PI staining assay showed that treatment with 40 μM of HPOB resulted in a moderate increase in the ratio of early and late apoptotic RPMI-8226 cells (Figure 3A,B). As shown in Figure 3C,D, U266 cells also displayed a high percentage of apoptotic cells aftertreatment with 40 μM of HPOB for 48 h. However, at the protein level, HPOB didnot activate the classical pathways involving Caspase3, PARP1 and Caspase9 (Figure 3E,F), which impliedthat HPOB induced MM cell death through other pathways as this was not acaspase-dependent apoptosis process. Therefore, our data implied that the inhibition of MM cell proliferation was closely associated with the cell cycle G1 phase arrest and cell apoptosis.

### 2.4. HPOB Overcame Bortezomib Resistance for MM Cells

**Figure 4:**
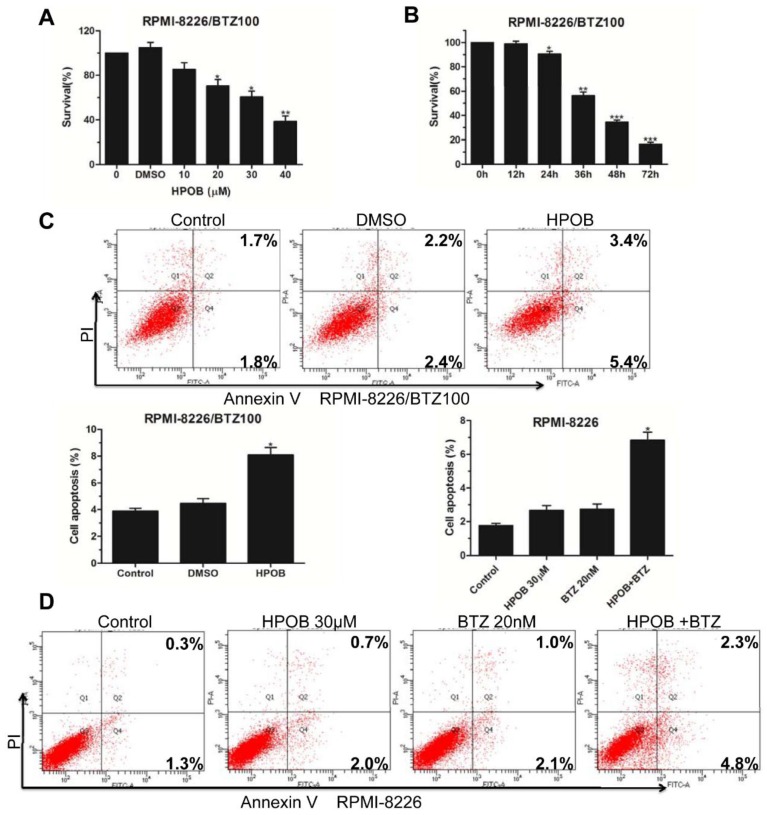
HPOB overcame the bortezomib resistance inMM cells. (**A**) RPMI-8226/BTZ100 cells were treated with different doses of HPOB for 48 h and analyzed by the CCK8 kit; (**B**) RPMI-8226/BTZ100 cells were treated by 40 μM of HPOB for different periods of time and cell survival was detected by the CCK8 assay; (**C**) Flow cytometry analysis of the percentage of apoptotic RPMI-8226/BTZ100 cells; (**D**) Flow cytometry analysis of the ratio of apoptosis in RPMI-8226 cells treated by HPOB and/or bortezomib. Error bars indicate mean ± SD. * *p* < 0.05, ** *p* < 0.01, *** *p* < 0.001.

As a novel therapeutic agent, bortezomib (BTZ) has been a great breakthrough in recent years [20,21]. However, about one-third of the patients withMM are resistant to bortezomib [22]. Thus, it isimportant to pursue new targeted molecular drugs to overcome bortezomib resistance. Herein, we chose 100 nM BTZ-resistant RPMI-8226 cell lines to conduct a CCK8 assay. The results showed that HPOB led to a decrease in the viability of RPMI-8226/BTZ100 cells in a dose- and time-dependent manner (Figure 4A,B). We further confirmed that the HPOB treatment induced the apoptosis of BTZ-resistant apoptosis compared with the DMSO control group (Figure 4C).

In the preliminary experiments, a lower dose of HPOB could not induce MM cell apoptosis, while thecombinations of 30 μM HPOB and 10 nM BTZ also did not trigger MM cell death evidently (data not shown). However, HPOB used in combination with 20 nM BTZ exhibited moderatepro-apoptotic functions (Figure 4D). Our results indicated that combining a lower concentration of HPOB with 20 nM of BTZ could sensitize multiple myeloma cells and HPOB could overcome bortezomib resistance for MM cells.

### 2.5. HPOB Promoted MM Cell Apoptosis via Transcriptional Activation of p21

**Figure 5:**
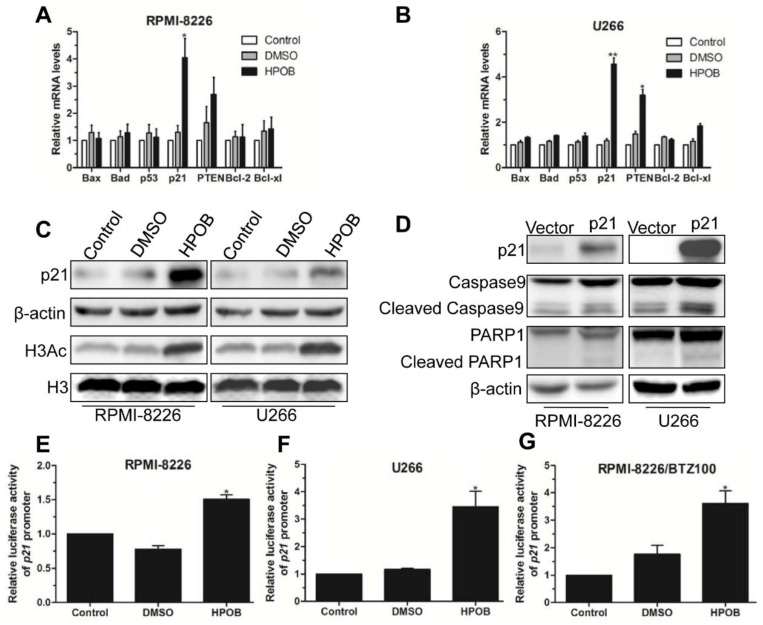
HPOB promoted MM cell apoptosis via transcriptional activation of p21. (**A**,**B**) Q-PCR analysis of apoptosis-associated factors in RPMI-8226 and U266 cells treated by 40 μM of HPOB for 48 h; (**C**) Western Blot analysis of the expression level of p21 and H3Ac in MM cells treated by HPOB. β-actin and H3 were used as internal controls, respectively; (**D**) Western Blot analysis of p21, Caspase9, PARP1 and corresponding cleaved forms in RPMI-8226 and U266 cells transfected with p21 plasmids; (**E**–**G**) Luciferase reporter gene assay analysis of p21 promoter activity in MM cells treated by 40 μM of HPOB for 48 h. Error bars indicate mean ± SD. * *p* < 0.05, ** *p* < 0.01.

To further elucidate the mechanism of HPOB-mediated cell death, we first detected the expression of apoptosis-associated factors by Q-PCR. The results showed that HPOB treatment led to a significant increase inp21 expression at the mRNA level in RPMI-8226 and U266 cells (Figure 5A,B). Subsequently, we found that HPOB increased the levels of p21 proteins in RPMI-8226 and U266 cells evidently (Figure 5C). As a HDAC inhibitor, HPOB’s function to regulate gene expression may rely on the change inhistone H3 and H4 acetylation modification. Therefore, we treated MM cells using an appropriate concentration of HPOB and extracted the nuclear proteins. The Western Blot assay indicated an increase in H3Ac (Figure 5C), but not in H4Ac (data not shown). As expected, the ectopic expression of p21 also activated the Caspase9 and PARP1 proteins in RPMI-8226 and U266 cells evidently (Figure 5D), which are important apoptosis-associated markers. After this, we wanted to know whether HPOB regulated p21 promoter activity. Our luciferase reporter gene assay demonstrated that HPOB could enhance the transcription of p21 promoter-driven luciferase reporter in normal MM cells or bortezomib-resistant MM cells (Figure 5E–G). The above results indicated that the HDAC inhibitor HPOB induced MM cell death via transcriptional activation of p21.

## 3. Materials and Methods

### 3.1. Reagents and Cell Culture

HPOB was purchased from Selleck (Houston, TX, USA). Furthermore, the stock solution of HPOB was generated by dissolving the compound in dimethyl sulfoxide (DMSO; Sigma Aldrich, St. Louis, MO, USA). Multiple myeloma cell lines RPMI-8226 and U266 were purchased from the American Type Culture Collection (Manassas, VA, USA). RPMI-8226/BTZ100 was donated by Dr. Jacqueline Cloos (VU University Medical Center, Amsterdam, The Netherlands) [23]. All the cells were grown in RPMI1640 medium which was supplemented with 10% FBS, at 37 °C and 5% CO_2_.

### 3.2. Cell Viability Assay

Briefly, the indicated cells were seeded into 96-well plate, before thecells were treated with different concentrations of HPOB for 48 h. Cell viability was assessed using the CCK8 kit according to the standard protocol (Beyotime, Shanghai, China). Experiments were performed in triplicate independently.

### 3.3. Cell Cycle and Apoptosis Analysis

The cell cycle assay was performed as described previously [24]. For apoptosis, after treatment with HPOB for 48 h, the cells were washed and resuspended in the buffer with Annexin V-FITC and PI, provided in apoptosis detection kit (KeyGEN, Nanjing, China), according to the manufacturer’s instructions.

### 3.4. Western Blot

The indicated cells treated by HPOB were lysed in the RIPA buffer containing protease inhibitor. Furthermore, the experiment was conducted according to the reports previously [25]. The following antibodies were used: β-actin (Santa Cruz Biotechnology, Santa Cruz, CA, USA); Caspase3 and Caspase9 (Cell Signaling Technology, Beverly, MA, USA); H3, PARP1 and p21 (Proteintech); andH3Ac (Active Motif, Carlsbad, CA, USA).

### 3.5. Quantitative Real-Time PCR (Q-PCR)

The total RNA was extracted from the indicated cells using TRIzol reagent (Takara). Furthermore, 1.5μgof RNA was used as atemplate for cDNA synthesis according to the manufacturer’s instructions (Promega). After this, Q-PCR was performed for Actin, Bax, Bad, p21, PTEN, Bcl-2 andBcl-XL according to aprevious study [25].

### 3.6. Luciferase Reporter Gene Assay

The luciferase reporter gene plasmid pGL4.20-p21 was transfected into the corresponding cell lines. After 24 h, the cell lysates were subjected to luciferase analysis using the Promega detection kit as reported previously [26].

### 3.7. Statistical Analysis

All results are presented as means + SD of three independent experiments. Student’s t-test was used to assess the statistical significance, with * *p* < 0.05 and ** *p* < 0.01. All data were analyzed using the GraphPad Prism 5 (GraphPad Software, LaJolla, CA, USA).

## 4. Conclusions

Epigenetic regulation is closely associated with the progressionof multiple myeloma. In recent years, emerging HDAC inhibitors have been approved for treatment of various tumors. In this study, the effect of the HDACinhibitor HPOB as a potential anti-tumor agent on the proliferation and apoptosis of MM cells had been investigated in detail. Our results showed that HPOB decreased the survival of MM cells in a dose- and time-dependent manner. Additionally, HPOB caused the accumulation of MM cells in the G1 phase compared with DMSO control group. Interestingly, we found that HPOB could overcome bortezomib resistance for MM cells and combining HPOB with BTZ could further sensitize MM cells. Certainly, our data illuminated that HPOB-mediated cell death was associated with transcriptional activation of p21, which was associated with an elevated global H3Ac modification level. Therefore, HPOB could be a potential candidate for MM treatment and the combination of HPOB and bortezomib is a possible therapeutic strategy for relapsed and refractory MM.

## Abbreviations

MM	Multiple myeloma
HPOB	Hydroxamicacid-based small-molecule*N*-hydroxy-4-(2-[(2-hydroxyethyl)(phenyl)amino]-2-oxoethyl)benzamide
DMSO	Dimethylsulfoxide
BTZ	Bortezomib
HDAC	Histone deacetylase
H3Ac	Histone 3 acetylation
